# *Notes from the Field:* Use of Enhanced Surveillance to Detect Severe Group A *Streptococcus* Infections Among Persons Experiencing Homelessness — Missoula County, Montana, July 2024–September 2025

**DOI:** 10.15585/mmwr.mm7542a1

**Published:** 2026-10-29

**Authors:** Beth Hopkins, Ashley A. Meehan, Stephen G. Mugel, B-Rad Applegate, Sarah Black, Joe B. B. Silva, Jeffry Mutuc, Kirsten Edwards, Josy Jahnke, Pam Whitney, Michelle Mozer, Sopio Chochua, Victoria Doll, Erika Baldry, Jessica Lopeman, Cindy Farr, Christopher J. Gregory, Laura Williamson, Cedar L. Mitchell

**Affiliations:** ^1^Montana Department of Public Health and Human Services; ^2^Career Epidemiology Field Officer Program, CDC; ^3^Epidemic Intelligence Service, CDC; ^4^Division of Bacterial Diseases, National Center for Immunization and Respiratory Diseases, CDC; ^5^Missoula Public Health, Missoula, Montana.

**Summary**

**What is already known about this topic?**

The incidence of invasive group A *Streptococcus* (GAS) infection among persons experiencing homelessness is approximately 100 times that among persons in the general population.

**What is added by this report?**

During July 27, 2024–September 20, 2025, a total of 69 GAS cases were identified in Missoula, Montana, using routine and enhanced disease surveillance; 39 (57%) cases (including three reinfections) occurred among 36 persons experiencing homelessness; 18 (46%) of these cases were identified through enhanced surveillance activities. Enhancing surveillance data to capture housing information improved homelessness classification among GAS patients and enhanced detection of GAS cases.

**What are the implications for public health practice?**

The disproportionately high incidence of GAS infection among persons experiencing homelessness that was missed by routine surveillance activities highlights areas for improving early case detection to prevent severe disease, case classification, and opportunities to prevent further spread.

Group A *Streptococcus pyogenes* (GAS) infections cause diverse clinical manifestations, including noninvasive disease such as skin or wound infections and invasive disease, which can result in severe illness and death (*1*). Skin breakdown from wounds, including blunt or penetrating trauma, burns, environmental exposure, and pressure ulcers, is common among persons experiencing homelessness and can contribute to invasive infections (*2*). In this population, the incidence of invasive GAS (iGAS)* disease is approximately 100 times that of the general population (*3*). Because of the potential for iGAS to cause severe disease, iGAS became a reportable condition in Montana on July 27, 2024 (*4*).

During November 2024–August 2025, routine disease surveillance by the Montana Department of Public Health and Human Services (DPHHS) detected 20 cases of invasive and noninvasive GAS infection epidemiologically linked to two homeless shelters in Missoula and raised concern about potential increased infections among persons experiencing homelessness. DPHHS requested epidemiologic assistance (Epi-Aid) from CDC to conduct enhanced surveillance and characterize GAS infections among persons experiencing homelessness in Missoula. Written consent was obtained from local health care facilities to review records. This activity was reviewed by CDC, deemed not research, and conducted consistent with applicable federal law and CDC policy.^†^

## Investigation and Outcomes

### Enhanced GAS Surveillance

In September 2025, enhanced disease surveillance activities were initiated to improve detection of GAS infections and systematically capture additional epidemiologic data, including housing status. Enhanced surveillance activities included 1) obtaining laboratory reports and medical records directly from local health care facilities, 2) abstracting clinical and epidemiologic information, and 3) sequencing available GAS isolates using whole-genome sequencing methods at the Utah Public Health Laboratory and CDC. The surveillance period was extended retrospectively to capture all GAS cases during July 27, 2024–September 20, 2025. Case definitions were developed for GAS infection (iGAS, severe noninvasive GAS, and nonsevere noninvasive GAS)^§^ and for homelessness (confirmed or probable).^¶^

### Characteristics of GAS Infections

Sixty-nine GAS cases were identified (Figure), 39 (57%) of which (including three reinfections)** occurred among 36 persons with confirmed or probable homelessness. Among the 39 cases of GAS infection among persons experiencing homelessness, eight were identified through extension of the surveillance period, and 10 were identified through other enhanced surveillance methods, including six from directly obtained laboratory reports and four from housing information in medical records. Among all 39 cases of GAS infection in persons experiencing homelessness, 34 (87%) required hospitalization, including the 15 (38%) iGAS infections; one iGAS infection resulted in death. Among the three reinfections among persons experiencing homelessness, all three patients had invasive disease for both their initial infection and their reinfection; two patients were hospitalized for both of their infections, whereas one patient was hospitalized for their initial infection only.

**FIGURE:**
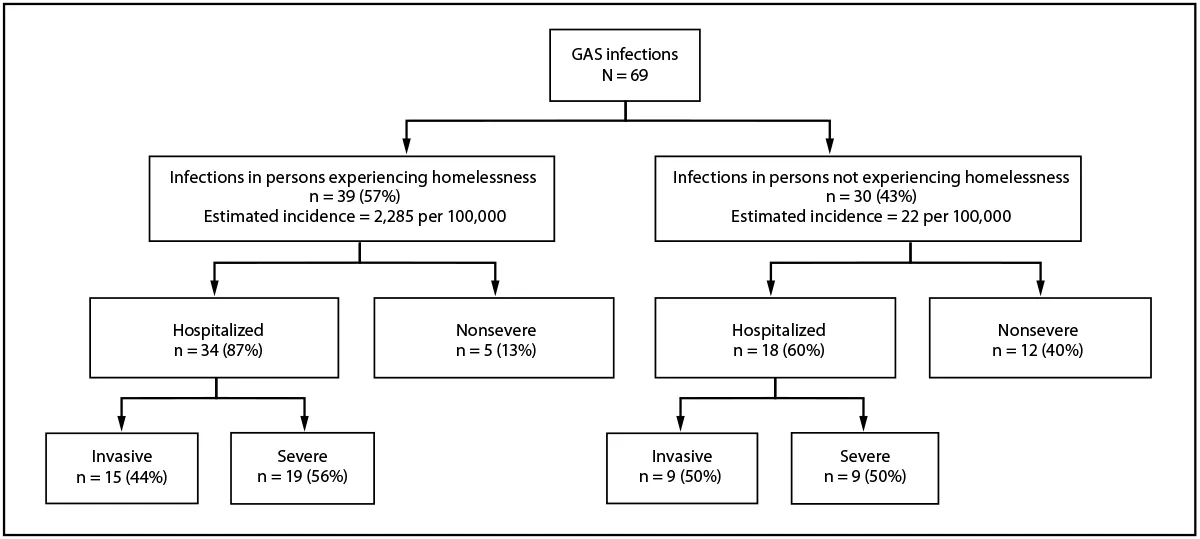
Number and percentage of group A *Streptococcus* infections* among persons experiencing homelessness^†^^,^^§^ and not experiencing homelessness, by hospitalization status of patients and severity of infection^¶^ — Missoula County, Montana, July 27, 2024–September 20, 2025 **Abbreviation:** GAS = group A *Streptococcus*. * All numbers refer to cases of GAS infection. Invasive GAS was defined as a GAS-positive culture from a normally sterile body site, streptococcal toxic shock syndrome, or necrotizing soft tissue infection. Severe GAS was defined as a GAS*-*positive culture (excluding throat culture) isolated from a nonsterile site (e.g., wound) in a person who was hospitalized or was recommended to be hospitalized but who left against medical advice. Nonsevere GAS was defined as a GAS-positive culture (excluding throat culture) isolated from a nonsterile site in a person who was not hospitalized or was not recommended to be hospitalized. ^†^ Housing status was classified as homeless if a person had documentation of service use in the Homeless Management Information System, documentation of homelessness in their medical record (e.g., history of illness, nursing notes, social history notes, or discharge notes), or a had an address associated with a homeless service provider or homeless shelter. ^§^ The 39 infections occurred among 36 persons and included three cases of reinfection. Among the three reinfections among persons experiencing homelessness, all three patients had invasive disease for both the initial infection and the reinfection. Two patients were hospitalized for both of their infections, whereas one patient was hospitalized for the initial infection but not the reinfection. ^¶^ One of the 15 persons experiencing homelessness who was hospitalized with invasive GAS died.

Thirty (43%) of the 69 cases of GAS infection were identified among persons not experiencing homelessness; no reinfections occurred among this group. Eighteen (60%) infections resulted in a hospitalization, including nine (50%) iGAS infections. During the surveillance period, the estimated incidence of GAS infections among persons experiencing homelessness (2,285.2 per 100,000 person-years) was 106.1 times (95% CI = 65.9–170.7) that in Missoula County’s general population (21.5 per 100,000 person-years).^††^

At the time of diagnosis, wounds or skin breakdown were present in 34 (87%) of the 39 cases of GAS infection among persons experiencing homelessness. History of substance use was common (27; 69%), and injection drug use, a known iGAS risk factor (*3*), was documented in 10 (26%) patient encounters. Data from 23 isolates revealed six M protein gene (*emm*) types,^§§^ suggesting genetic diversity among infections and multiple transmission chains.

## Preliminary Conclusions and Actions

Persons experiencing homelessness experience a disproportionately high incidence of GAS infection; therefore, early detection in this population to prevent severe disease remains a high priority (*3*). The identification of 18 (46%) additional GAS cases among persons experiencing homelessness, through enhanced surveillance or retrospective extension of the surveillance period, highlighted the usefulness of enhanced surveillance for an emerging public health concern among a population at high risk for infection. Estimating the incidence of GAS among persons experiencing homelessness remains a challenge because of differential barriers to accessing health care and case ascertainment by housing status, dynamic population denominators and limited enumeration data, and incomplete classification of housing status; thus, these findings likely underestimate the true incidence of GAS infection among persons experiencing homelessness. DPHHS now routinely collects housing status data during iGAS investigations to improve disease detection in this population (*5*) and facilitate prompt public health action to prevent further spread. The department is also exploring thresholds for increasing and decreasing enhanced surveillance activities to respond to GAS cases and other emerging public health events.

## Footnotes

* iGAS was defined as a GAS-positive culture isolated from a normally sterile body site, streptococcal toxic shock syndrome, or necrotizing soft tissue infection.

† 45 C.F.R. part 46, 21 C.F.R. part 56; 42 U.S.C. Sect. 241(d); 5 U.S.C. Sect. 552a; 44 U.S.C. Sect. 3501 et seq.

§ GAS severity was classified as 1) invasive GAS, defined as a GAS-positive culture from a normally sterile body site, streptococcal toxic shock syndrome, or necrotizing soft tissue infection; 2) severe noninvasive GAS, defined as a GAS-positive culture (excluding throat culture) isolated from a nonsterile site (e.g., wound) in a person who was hospitalized or was recommended to be hospitalized but who left against medical advice; or 3) nonsevere noninvasive GAS, defined as a GAS-positive culture (excluding throat culture) isolated from a nonsterile site in a person who was not hospitalized or was not recommended to be hospitalized.

¶ 1) Confirmed homelessness: documentation of service use in the Homeless Management Information System, or documentation of homelessness in a patient’s medical record (e.g., history of illness, nursing notes, social history notes, or discharge notes); 2) Probable homelessness: an address associated with a homeless service provider or homeless shelter without documentation of homelessness in the medical record.

** Reinfections were defined as reisolation of GAS in a person with a documented previous GAS infection ≥30 days after the previous isolation.

†† GAS incidence among persons experiencing homelessness was estimated at 2,285.2 per 100,000 person-years for 39 cases among a population denominator of 1,484 persons derived from the Missoula Community Houselessness Strategy 2024 annual estimate. GAS incidence among persons not experiencing homelessness was estimated at 21.5 per 100,000 person-years for 30 cases among a population denominator of 121,062 persons in Missoula County, obtained by subtracting the estimate among persons experiencing homelessness (1,484) from the total Missoula County U.S. Census Bureau 2024 population projection (122,546). The surveillance period was 1.15 years.

§§ Group A streptococci are classified into >275 *emm *types, based on sequence analysis of part of the cell surface M (virulence) protein. Among the 23 isolates, the dominant *emm* types were *emm* 49 (detected in nine isolates) and *emm* subtype 58.2 (detected in 10 isolates). In the remaining four isolates, one *emm* type (53, 76, 85, or 92) was detected in each isolate.
